# Consulting doctors online after offline treatment: investigating the effects of online information on patients' effective use of online follow-up services

**DOI:** 10.3389/fpubh.2024.1375144

**Published:** 2024-04-09

**Authors:** Shuhui Han, Lun Li

**Affiliations:** ^1^School of Management and Economics, Beijing Institute of Technology, Beijing, China; ^2^School of Management, Fudan University, Shanghai, China

**Keywords:** online follow-up services, online health communities, doctors' knowledge contribution, patient feedback, effective use

## Abstract

**Introduction:**

The use of online follow-up services (OFUS) is becoming an increasingly important supplement to hospital care. Through OFUS, patients can find their doctors in online health communities (OHCs) and receive remote medical follow-ups after hospital treatment. However, the rate of effective use of OFUS by current patients is still low, and there is an urgent need for research to investigate the online information factors that affect patients' effective use of OFUS.

**Methods:**

Based on the elaboration likelihood model (ELM) of persuasion and an analysis of a panel dataset including 3,672 doctors in a leading OHC in China, this study explores how online information from doctors' knowledge contributions and patient feedback influences patients' effective use of OFUS.

**Results:**

The results show that both doctors' knowledge contributions and patient feedback positively influence patients' effective use of OFUS. Doctors' paid knowledge contributions and patients' paid feedback have stronger persuasive effects than doctors' free knowledge contributions and patients' free feedback, respectively. Moreover, there is a substitutional relationship between doctors' paid and free knowledge contributions and between patients' paid and free feedback in influencing patients' effective use of OFUS.

**Discussion:**

The findings of this study suggest that OHC platforms and healthcare providers should account not only for the persuasive effects of doctors' knowledge contributions and patient feedback but also for influential differences and relationships between the types of doctors' knowledge contributions and patient feedback to better persuade patients to effectively use OFUS.

## Introduction

Medical follow-ups are important for improving patients' health outcomes and medical experiences (1). Through medical follow-ups, doctors can monitor patients' health status, answer their questions, modify their treatment plan and medicine regimen, and more. Traditionally, offline face-to-face communication and telephone communication between doctors and patients have been the primary means of medical follow-ups (2). However, the travel costs and time demands of participating in offline follow-ups have led to a high patient no-show rate for follow-ups, which has been a persistent threat to public health (3). Although telephone follow-up communication may reduce the costs for patients, it does not allow for immediate access to medical records and test results or proper medical record-keeping, which can lead to incomplete discussions and discontinuity of care. The scheduling and coordination of telephone appointments can also pose challenges that potentially inconvenience both patients and providers (4). In recent years, the rapid development of online health communities (OHCs) in China has made it possible for doctors to provide medical follow-up services through online channels, which could reduce costs for patients and improve medical follow-up efficiency and outcomes (5).

Several OHCs (e.g., haodf.com in China, Speedoc in Singapore) have already introduced online follow-up services (OFUS) (5). With this option, a patient can first visit a doctor through an offline channel, such as a hospital or clinic, for an in-person consultation, examination, or treatment with the doctor. After the offline visit, the patient can utilize the doctor's OFUS, through which the doctor can continue serving the patient online in regard to subsequent health recovery and future plans. Thus, through the OFUS, the patient can still communicate with the doctor who assisted them in the hospital or clinic. In the usage mode of OFUS on the haodf.com platform, for instance, the patient first scans a QR code to find their doctor online. Then, they enter their demographic information, fill in the offline treatment date, and upload certifications of offline treatment (e.g., medical records and receipts) with the doctor. Once the doctor verifies the patient's identity and the relevant documents, the patient can use their OFUS to communicate with the doctor and receive appropriate ongoing health follow-up management. Online follow-up services present significant advantages as an important mode of online doctor-patient communication. For example, asynchronous online communication can facilitate a flexible and convenient exchange of information between a patient and their doctor without time- or location-related restrictions (6). Moreover, patients and doctors can easily access medical histories, lab results, imaging reports, and treatment plans, which could foster more informed and meaningful follow-up communication (7).

Despite these advantages, there is still a low adoption rate of OFUS by patients in China. More importantly, the majority of current adopters of OFUS do not use OFUS effectively to manage their health or even engage in any follow-up communication with their doctors. Patients may be hesitant to use OFUS because they lack trust in this emerging medical follow-up service, especially due to concerns about health privacy and data security on OHC platforms (8). However, previous research on health information technology (IT) suggests that only the effective use (and not the simple adoption) of IT-based health portals can improve patients' health outcomes (9). To better understand this phenomenon, it is crucial to investigate the online information factors that influence patients' effective use of OFUS. Such inquiries can yield valuable insight for optimizing the communication strategies of OHC platforms and healthcare providers to encourage positive use of OFUS by patients.

As an emerging medical follow-up model, OFUS have not been carefully examined in the literature. To the best of our knowledge, only one study by Li et al. (10) has explored the effects of several online information factors on patients' use of doctors' OFUS. However, their study focused on whether patients registered for doctors' OFUS (i.e., simple adoption behaviors of OFUS) rather than patients' effective use behaviors of OFUS, and it considered a limited range of persuasive online information factors. To fill the research gaps, the present study aims to determine which online information on OHC platforms influences patients' effective use of OFUS. We adopt the elaboration likelihood model (ELM) of persuasion as the theoretical basis to explore how patients process online information cues when making decisions relating to the effective use of OFUS. The ELM suggests that individual attitudes and behaviors are influenced by persuasive information through two routes: the central route and the peripheral route (11). When processing information through the central route, individuals must devote considerable cognitive effort to think critically about issue-related arguments and assess the arguments' quality. By comparison, peripheral route processing requires less cognitive effort because it relies on simple heuristic information (e.g., source credibility). In practice, individuals are often affected simultaneously by central and peripheral cues of persuasive information (12).

Following previous research using the ELM (10, 13), this study considers the information from doctors' knowledge contributions as a central cue and the information from patient feedback as a peripheral cue. In most OHCs, the knowledge contributions of doctors include both paid and free knowledge contributions (14). Both types are based on the doctors' medical expertise, which directly relates to the doctors' service quality; thus, patients use the central route to process them. Similarly, patient feedback on most OHC platforms has both paid and free forms, but it is provided by patients to doctors (15). This feedback is considered an important source of electronic word of mouth (eWOM) that reflects a doctor's credibility (13). Hence, patients process patient feedback through the peripheral route. In view of the above discussion, we aim to answer the following research questions to establish how the effective use of OFUS by patients is influenced by the different types of online information.

(1) How do doctors' knowledge contributions, including both paid and free knowledge contributions, influence patients' effective use of OFUS? Which one has a stronger effect? Do they have a substitutional relationship in influencing patients' effective use of OFUS?

(2) How does patient feedback, including both paid and free patient feedback, influence patients' effective use of OFUS? Which one has a stronger effect? Do they have a substitutional relationship in influencing patients' effective use of OFUS?

The answers to these questions can clarify how different types of online information influence patients' effective use of OFUS. Such knowledge could assist OHC platforms and OFUS providers in designing better information communication strategies that persuade patients to positively use OFUS.

## Literature review

### Medical follow-ups

The body of research on medical follow-ups can be classified according to the mode of service delivery. The first stream of literature mainly centers on medical follow-ups rendered through *offline face-to-face visits*. Numerous studies have demonstrated the value of these offline follow-ups for mitigating patient morbidity and mortality (16), curbing readmission rates (17), and improving patient satisfaction (18). Moreover, some research has explored how intervention strategies, such as email reminders (19), telephone reminders (20), and short message services (SMS), could increase patient adherence to offline medical follow-ups (21). Related studies have found that these intervention strategies can reduce non-attendance among patients who require long-term medical follow-ups (22).

The second stream of literature focuses on medical follow-ups conducted through telephone communication (i.e., telephone follow-ups). These studies have investigated the effectiveness of this means of follow-up in different contexts, such as the improvement of patients' medication compliance (23), adherence to therapeutic regimens (24), post-discharge health outcomes (25), and patient satisfaction (26). Furthermore, research has compared telephone follow-ups to traditional offline follow-ups. For example, Booker et al. (27) found that telephone follow-ups were amenable to the majority of patients as a substitute for offline follow-ups. Multiple studies have empirically identified the benefits of telephone follow-ups, which include convenience (2) and lower costs (28).

As an emerging channel of medical follow-ups, OFUS is becoming increasingly popular and gaining widespread attention. However, existing research on medical follow-ups has predominantly addressed traditional offline face-to-face and telephone follow-ups, and there is a noticeable research gap concerning patients' use of OFUS. Given the importance of OFUS in the digital healthcare ecosystem, its usage deserves further exploration.

### Online information and patient decisions

The OHC around doctor-patient communication has emerged as a transformative solution in the healthcare industry to bridge the gap between medical providers and patients through digital channels (29). Doctors on OHC platforms offer multiple healthcare services, such as online consultation services, offline appointment services, and OFUS, that cater to patients' various healthcare needs (30). The dynamic engagement of both doctors and patients in OHCs has led to a prolific generation of online health information in two main forms: doctors' knowledge contributions and patient feedback information (31).

Doctors' knowledge contributions encompass a wealth of medical knowledge that healthcare professionals provide to patients. Knowledge contributions can be paid or free (32). With paid knowledge contributions, doctors furnish specialized insights, treatment recommendations, and medical expertise for patients who seek personalized guidance by purchasing online consultation services (33). With free knowledge contributions, doctors add to the OHC platform's reservoir of health knowledge by sharing health knowledge information at no cost (34). Such contributions include the dissemination of health science education articles, which can empower patients with information about their conditions and wellness practices.

Patient feedback refers to the evaluations and comments provided by patients based on their personal experiences with medical consultations. This feedback is a significant component of eWOM (35), as it informs prospective patients about a doctor's competence, bedside manner, and overall service quality (36). Like doctors' knowledge contributions, patient feedback can be paid or free (15). Paid feedback is a distinctive service feedback mechanism on OHC platforms that allows patients to purchase virtual gifts (e.g., digital cards) conveying their gratitude and appreciation for the guidance and care of their doctor. Meanwhile, free feedback is given through non-monetary actions, such as writing online reviews, posting thank-you letters, and casting votes. Although no financial transaction is involved, free feedback also reflects patient satisfaction with the medical services of a doctor.

Given the importance of online healthcare services, previous research has extensively investigated online information factors influencing patients' use of doctors' online healthcare services. However, extant research has mainly centered on patients' use decisions regarding online consultation services (i.e., how patients select a doctor for online consultations) (13, 37, 38) and offline appointment services (i.e., how patients select a doctor for offline appointments) (39–41) and has not fully investigated patients' use of doctors' OFUS, especially their effective use behaviors in this context (10).

Only one study has examined the role of doctors' online service quality (e.g., paid knowledge provision) and eWOM (e.g., free patient feedback) in the adoption of OFUS by patients after offline treatment (10). While that study provides insight into patients' adoption of OFUS, it mainly emphasized decision-making and overlooked patients' effective use behaviors with OFUS. Maintaining a continuum of care and improving patients' health outcomes requires effective use (rather than simple adoption) of OFUS. The aforementioned study did not comprehensively consider the broader spectrum of online information (e.g., doctors' free knowledge contributions, patients' paid feedback) that potentially influences patient engagement with OFUS. To enhance the effectiveness and utilization of OFUS as an important element of the digital healthcare ecosystem, it is imperative to understand the impacts of a wider array of online information on patients' effective use of OFUS.

## Theoretical framework and research hypotheses

### The elaboration likelihood model

The ELM is a widely used theory of persuasion (11). This model offers a compelling framework for how people process and respond to persuasive messages (42). According to the ELM, people's attitudes and behavioral changes are influenced by persuasive information through two routes of information processing—the central route and the peripheral route (11)—which involve different levels of elaboration. Through the central route, individuals carefully scrutinize a message, evaluate its argument, and consider the relevant information. Processing through the central route is characterized by thoughtful analysis, critical thinking, and a focus on the content of the message. This type of processing leads to lasting attitude changes and a stronger persuasive effect. By comparison, the peripheral route requires less cognitive resources, as it relies on heuristic or peripheral cues, such as source credibility, attractiveness, or emotional appeal, in making judgments about a message. Peripheral processing involves quicker, less intensive evaluations and can produce temporary attitude adjustments that are more susceptible to change. In theory, people can process a message exclusively via the central route or the peripheral route, but empirical observations have indicated that, in reality, message elaboration often takes place at a moderate level that utilizes both routes (12).

The ELM has been applied to study a wide range of health-related topics, such as health behavior changes (43, 44), mobile health app adoption (45), health information adoption (46), and the use of health services (13). Studies using the ELM have reported that both central cues and peripheral cues can simultaneously influence health attitudes and behavioral changes of individuals. For example, in a study of online health service use, Cao et al. (13) applied the ELM to explore the effects of central cues (e.g., doctors' paid knowledge contributions) and peripheral cues (e.g., patient votes) on patients' selection of online consultation services. In the present study, the ELM similarly provides a conceptual foundation to understand how the online information cues of doctors' knowledge contributions and patient feedback influence patients' effective use of OFUS.

**Figure 1:**
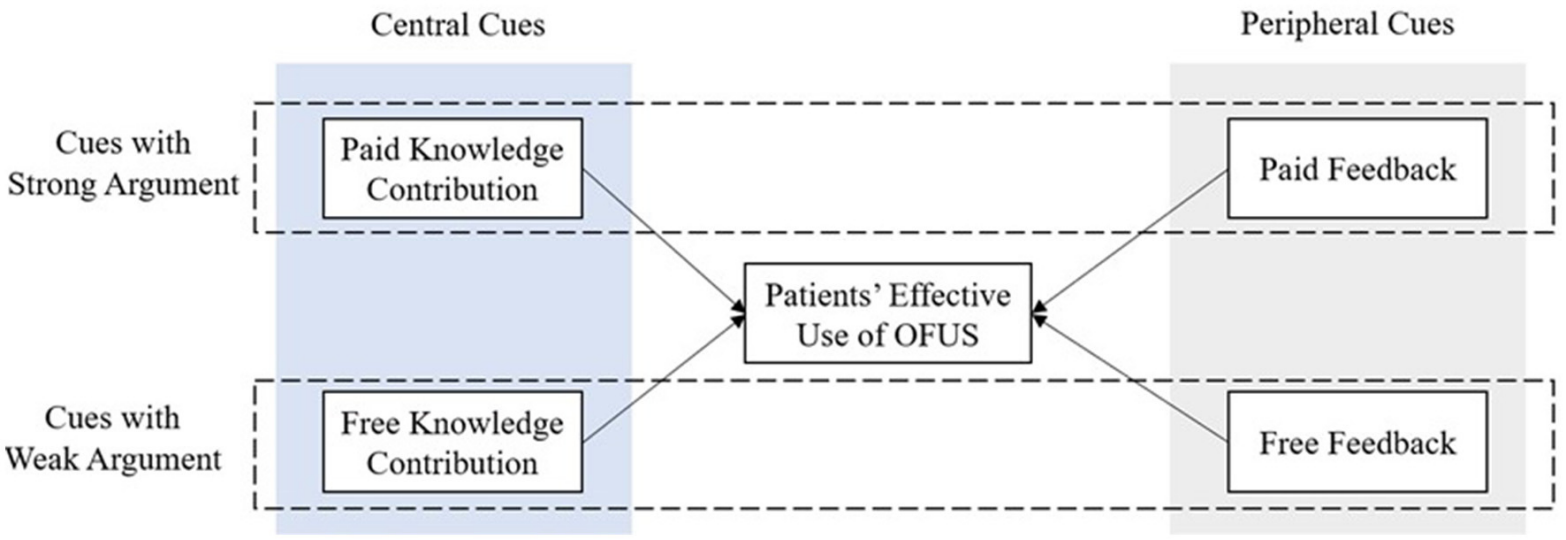
Research framework based on the ELM.

The following sections explain why doctors' knowledge contributions (the central cue) are processed through the central route, while patient feedback (the peripheral cue) is processed through the peripheral route. Subsequently, we elaborate on the research hypotheses concerning the predictive relationship between information from doctors' knowledge contributions or patient feedback and patients' effective use of OFUS (see Figure 1).

### Central cues: doctors' knowledge contributions

In the current study, we regard doctors' knowledge contributions (both paid and free) as the central route cue because they directly relate to doctors' online service quality. As discussed above, doctors' knowledge contributions are generally supported by the medical expertise, research, and experience of the doctors (33). According to the ELM (11), this information is a central cue because its expert-driven, authoritative, and information-rich nature requires patients to use more cognitive processing effort. Thus, when individuals encounter information in the form of doctors' knowledge contributions, they are more likely to engage in central-route processing, which involves scrutinizing the content of the information, critically evaluating its quality, and assessing its relevance to their own health concerns. This concept aligns with the idea of systematic processing, wherein an individual carefully evaluates the argument presented in a message (47).

In the literature on persuasion and communication, message quality is consistently considered a primary aspect (48, 49). In the ELM, argument quality pertains to how readers subjectively perceive the argument of a persuasive message and whether they deem it robust and compelling or feeble and fallacious (48). An individual forms their attitude toward a message by carefully deliberating the merits and quality of the argument it presents. The argument quality predominantly shapes individuals' attitudes toward a message by prompting thoughtful consideration of the presented argument's merits. Similarly, in the online healthcare context, service quality is considered a significant antecedent of patients' use of doctors' online services (10, 13). Since patients usually believe that doctors who provide higher-quality service will be more capable of diagnosing and treating their disease, they tend to be most concerned with a doctor's service quality. By this logic, in the context of the present study, patients should more likely to effectively use a doctor's OFUS if that doctor has a higher online service quality. Information about a doctor's knowledge contributions is an important central cue that patients can carefully process through the central route to assess the doctor's service quality. A high level of knowledge contributions by a doctor can better persuade their offline patients to use their OFUS after receiving offline treatment. Accordingly, we propose the following hypothesis:

H1: *A doctor's knowledge contributions (both paid and free) will have a positive influence on patients' effective use of OFUS*.

While both paid and free knowledge contributions by doctors are significant central cues, they may differ in their argument strength in persuasive communication. Argument strength is a significant concept in persuasion (50, 51). It refers to the quality, strength, and persuasiveness of an argument employed in persuasive communication. Petty and Cacioppo (11) have stated that the strength of an argument is foundational to its quality, as the “strong version of the message” offers compelling evidence to support a point of view, whereas the “weak version of the message” relies more heavily on quotations, personal opinions, and illustrative examples.

In our study, the paid knowledge contribution information stems primarily from doctors' one-on-one paid consultation services for patients. This information encompasses detailed medical insights with higher specialization, depth, and specificity (33). Thus, it can provide patients with a more profound impression of the doctor's professional service quality (13). In contrast, free knowledge contribution information often prioritizes accessibility and simplicity with the aim of enhancing patients' health literacy (34). These contributions mainly manifest as sharing health popular science articles or content (e.g., illustrative examples) targeting a broader audience and emphasizing relatability and ease of understanding (52). While free knowledge contribution information provides valuable insight into a doctor's medical expertise, it may not showcase the same level of detailed medical expertise seen in paid knowledge contribution information. Thus, the argument strength of free knowledge contributions should be weaker than that of paid knowledge contributions. On this basis, we consider a paid knowledge contribution to be a central cue with a strong argument (i.e., a strong central cue) and a free knowledge contribution to be a central cue with a weak argument (i.e., a weak central cue).

Extant research suggests that a message with a strong argument has a more persuasive effect than a message with a weak argument (11, 44). Strong arguments tend to elicit a higher number of favorable or unfavorable thoughts about a phenomenon and fewer counter-arguments, whereas weak arguments are less thought-provoking and more susceptible to rebuttals (11, 53). Thus, in the present study, doctors' paid knowledge contributions should generate a stronger persuasive effect on patients' decisions regarding the effective use of OFUS compared to free knowledge contributions. Accordingly, we propose the following hypothesis:

H2: *Doctors' paid knowledge contributions will have a stronger effect on patients' effective use of OFUS compared to free knowledge contributions*.

In addition, according to the ELM (11), patients might need to allocate more cognitive resources to carefully process and evaluate paid knowledge contribution information compared to free knowledge contribution information. Due to the limitation of patients' cognitive resources when processing information through the central route, an increase in the processing of paid knowledge contributions may correspond to a decrease in the processing of free knowledge contributions. Thus, the persuasive effect of paid knowledge contribution information, a strong central cue, is likely to substitute that of free knowledge contribution information, a weak central cue. Accordingly, we hypothesize the following:

H3: *Doctors' paid knowledge contributions and free knowledge contributions will have a substitutional relationship in influencing patients' effective use of OFUS*.

### Peripheral cues: patient feedback

This study considers patient feedback as the peripheral route cue. As discussed above, patient feedback includes paid feedback in the form of virtual gifts as well as free feedback, such as online reviews, thank-you letters, and votes. This feedback information can be seen as emotional and heuristic cues because it represents expressions of gratitude and appreciation from patients to doctors (31, 35). Subsequent patients can quickly scan this information and assess the doctors' service quality based on these emotional cues without engaging much cognitive effort. Therefore, according to the ELM (11), patient feedback on OHC platforms would be processed by patients via the peripheral route due to its emotionally expressive nature and ease of processing.

Most extant research regards consumer feedback (e.g., online consumer reviews) as an important form of eWOM for service providers (54, 55). In the eWOM communication literature, eWOM represents a prominent signal of the trustworthiness of service providers (56). The ELM literature suggests that source credibility information, such as eWOM, positively influences individuals' attitudes via the peripheral route (57). Similarly, patient feedback, as eWOM for doctors, may influence patients' trust in the OFUS of doctors through the peripheral route (13). Especially when patients lack the motivation or ability to deeply process information from doctors' knowledge contributions, patient feedback can offer a “shortcut” in decision-making by shaping patients' perceptions of and attitudes toward the online service quality of doctors. Patients may be more likely to engage in actively effective use of a doctor's OFUS if that doctor has received more free or paid feedback from other patients. In other words, doctors receiving more free or paid feedback can motivate their offline patients to effectively use their OFUS. Accordingly, we hypothesize the following:

H4: *Patient feedback, including paid and free feedback, will have a positive influence on patients' effective use of OFUS*.

Although paid and free feedback from patients both fall within the realm of peripheral cues, they might have different levels of argument strength and thus result in heterogeneous persuasive effects on subsequent patients. This difference can be explained by the ELM's peripheral route and the underlying psychological mechanisms. Commitment and investment are significant factors related to the argument strength of patient feedback. When patients opt to give paid feedback (e.g., virtual gifts), they not only convey appreciation but also dedicate financial resources to express their satisfaction (58). This financial investment indicates a deeper level of commitment to and involvement in the doctor-patient relationship, which signals that the patient highly approves of the doctor's medical services (15). This commitment acts as a robust peripheral cue that contributes to the argument strength of paid feedback. The perceived credibility of paid feedback can also derive from the belief that individuals spend money judiciously, especially when publicly endorsing a medical service. The inherent assumption is that patients would not invest financially unless they were genuinely satisfied with the doctor's medical services (59). This assumption enhances the credibility of paid feedback. While free feedback also holds value, it might have a comparatively lower argument strength due to the absence of a direct investment. Because free feedback does not involve a financial transaction, it may be perceived as easier to offer and therefore less indicative of a deep-seated belief in the doctor's service quality (15). In this regard, we consider paid feedback to be a peripheral cue with a strong argument (i.e., a strong peripheral cue) and free feedback to be a peripheral cue with a weak argument (i.e., a weak peripheral cue). Thus, we propose the following:

H5: *Paid feedback will have a stronger positive influence on patients' effective use of OFUS compared to free feedback*.

In addition, given the higher argument strength of paid feedback, it could potentially overshadow the impact of free feedback. Doctors who have received more paid feedback may have already generated positive eWOM that transfers a clear cue of their service quality to patients. In this case, more paid feedback might motivate patients to make a quick and relatively effortless decision, which would diminish the effects of information cues in free feedback. Extant research has found that paid feedback and free feedback have a substitutional relationship in influencing patients' selection of online consultations (15). Likewise, in regard to patients' effective use decisions about doctors' OFUS, we propose the following:

H6: *Paid feedback and free feedback will have a substitutional relationship in influencing patients' effective use of OFUS*.

## Methods

### Research context and data collection

**Figure 2:**
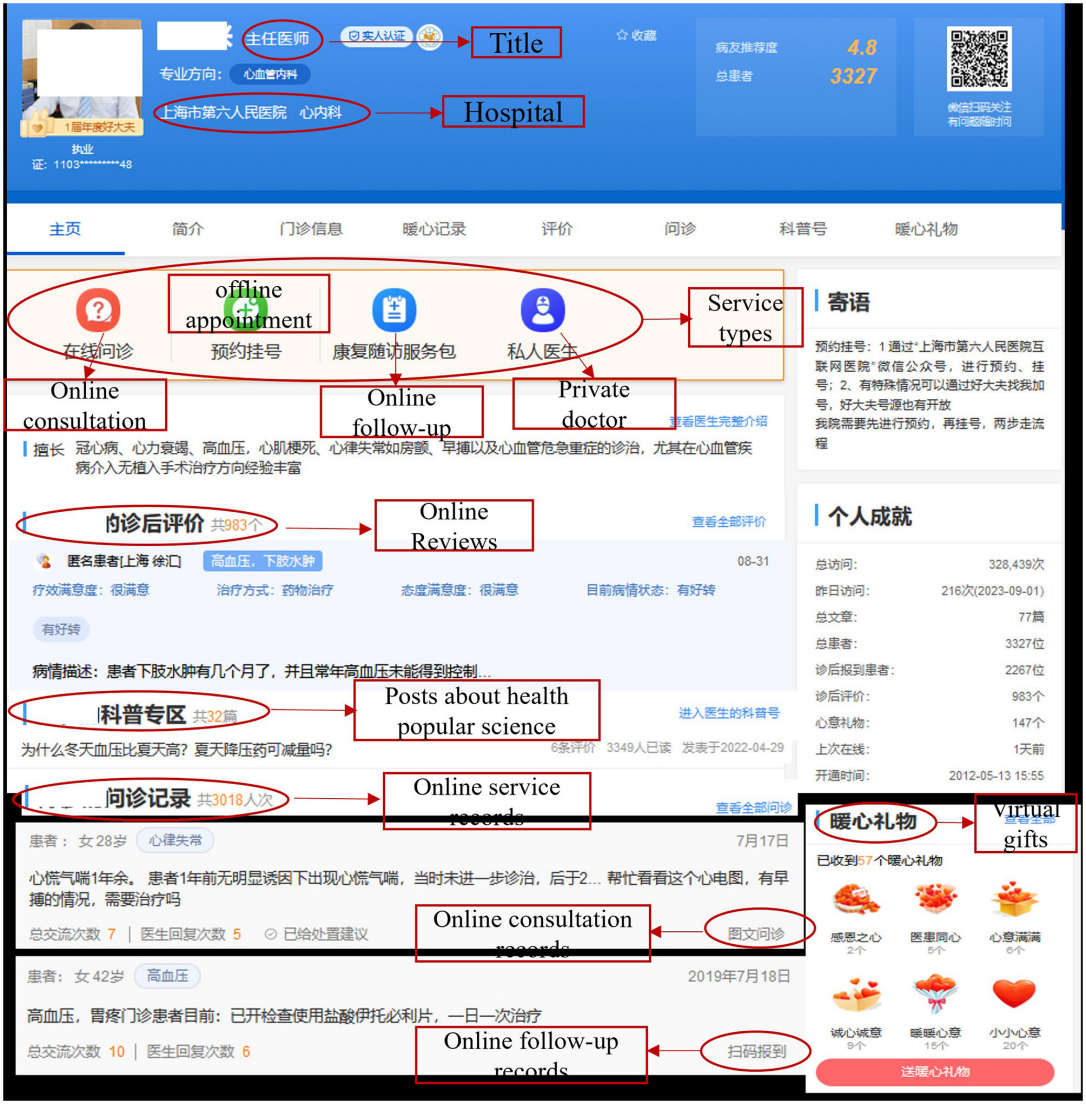
An example of a doctor's homepage.

Our research context is the leading Chinese OHC haodf.com. This OHC platform, like Zocdoc and Amwell in the United States, provides an online channel for patient-doctor communication. Doctors on this platform can provide several types of medical services for patients, such as online consultation services, offline appointment services, and OFUS. As of July 2023, over a quarter of a million doctors have registered with their real names on this platform and have served more than 84 million patients.^1^ Thus, the platform has a wealth of doctor-patient information. After registering on the platform, a doctor will set up a personal homepage displaying information such as a list of medical services they provide, posts about medical popular science shared by the doctor, online service records, patients' online reviews, and virtual gifts as well as a detailed profile containing the doctor's personal image, name, medical title, working hospital, specialty, medical skills, and outpatient information. This large volume of information offer a suitable research context to investigate the effects of different online information on patients' effective use of OFUS. Figure 2 displays an example of a doctor's homepage.

Since the online information on a doctor's homepage is publicly observable and accessible, we developed a Python program to crawl the above-mentioned information about doctors. Our research covered the period from January 2018 to January 2020. In our sampling of doctors, we mainly focused on those specialized in two specific chronic diseases, namely diabetes and coronary heart diseases, since chronic diseases often require regular and repeated treatment and follow-ups. To ensure the relevance of our analysis, we limited our dataset to only those doctors who had registered on the OHC platform prior to January 2018, the starting point of our observation window, and who provided at least one paid online consultation and one follow-up service during the observation window. Through this selection process, we identified a sample of 3,672 doctors. Subsequently, we constructed a panel dataset for the 25-month research period. The 1-month level is considered appropriate in the current context because patients' follow-up communication with doctors through OFUS may not occur immediately after their offline treatment. The final dataset consists mainly of each sampled doctor's basic characteristics (e.g., title, hospital, specialty, online tenure), paid online consultation records and online follow-up records, shared posts about medical popular science, and information about patients' online reviews, ratings, and virtual gifts. In the following section, we explain how we used this information to measure our research variables.

### Measurement

#### Patients' effective use of OFUS

Since each sampled doctor's OFUS records are accessible on the platform, we were able to observe the follow-up communication records. We noticed that many patients did not communicate with their doctor at all after adopting their OFUS. In view of this observation and previous research (9), we judged that a patient engaged in effective use of OFUS if they had at least one communication with their doctor after adopting their OFUS. Otherwise, we concluded that the patient adopted the OFUS but did not effectively use it. Given that the privacy protection policy of the platform prevents the observation of patient-level characteristics, we constructed our variables at the doctor-month level. Following similar studies (13, 31), we used the number of patients who effectively used a doctor's OFUS each month to measure patients' effective use of the OFUS for each sampled doctor.

#### Doctors' knowledge contributions

For each sampled doctor, *the doctor's paid knowledge contribution* was measured by the cumulative number of paid online consultation services that the doctor provided to patients, in line with previous research (10, 30). As discussed, doctors can charge certain fees for online consultation services for patients. Through paid online consultation services, doctors answer patients' medical questions. Thus, we considered the cumulative number of paid online consultation services provided by a doctor to be representative of the doctor's provision of health knowledge to patients and the doctors' medical expertise. *A doctor's free knowledge contribution* was measured by the cumulative number of posts about medical popular science that the doctor shared on their homepage (34, 52). Doctors usually voluntarily share posts about medical popular science on their homepages to broaden patients' basic knowledge. For example, one doctor shared posts addressing the questions, “Can diabetics eat fruit?” and “What are the dangers of high blood pressure?” Patients can read these posts to gain health knowledge without any extra financial cost. Thus, we considered the cumulative number of posts about medical popular science to reflect the free knowledge contribution of the doctor.

#### Patient feedback

For each sampled doctor, we measured *patients' paid feedback* as the cumulative number of virtual gifts that the doctor had received from patients, in accordance with previous research (15, 58). After an online consultation, a patient can buy a virtual gift to give to the doctor if they were very satisfied with the doctor's medical services. This virtual gift is similar to a digital card expressing appreciation for a doctor's service. Doctors can obtain monetary rewards corresponding to the price of these virtual gifts. Thus, the gifts can be regarded as a type of paid feedback from patients. Besides giving virtual gifts, patients on the platform can post gratitude letters to share feedback about the doctor's services. Thus, following previous research (15), we measured *patients' free feedback* as the cumulative number of gratitude letters that a doctor received from patients. Through gratitude letters, patients can provide text-based feedback about services (e.g., “Awesome doctor, everything I needed done was attended to quickly and generally cares about the wellbeing of the patient”). Unlike the virtual gifts, gratitude letters are free to write. Thus, we regarded these letters as a type of free feedback from patients. Notably, both the gratitude letters and the virtual gifts are associated with positive feedback about a doctor's service quality.

#### Control variables

Our control variables included two time-variant factors that might influence patients' use of a doctor's OFUS: the doctor's online rating and online tenure. The *online rating* of each doctor was measured by the doctor's cumulative average rating from patients (60). Online ratings reflect patient satisfaction and thereby influence the use decisions of subsequent patients. Meanwhile, a doctor's *online tenure* was measured by the number of days that had passed since the doctor created their personal homepage (10, 13). The online tenure reflects the doctor's experience with providing online services. A longer online tenure indicates more experience. Thus, we controlled for both online rating and online tenure to reduce confounding effects. The doctors' demographic characteristics (e.g., age, gender, title, hospital) were not included as control variables because the two-way fixed effects model based on panel data in the empirical analysis could control for these time-invariant variables (61). Table 1 displays the variable measurements and descriptive statistics.

**Table 1:** Variable definitions and descriptive statistics.

**Variables**	**Measurements**	**Mean**	**SD**
Patients' effective use of doctors' OFUS (*Effective*_*Use*_*it*_)	The number of patients who have effectively used a doctor's OFUS.	13.292	28.126
Paid knowledge contribution (*Paid*_*Contribution*_*it*_*)*	The cumulative number of paid online consultation services that a doctor provides to patients.	1,334.756	2,465.713
Free knowledge contribution (*Free*_*Contribution*_*it*_)	The cumulative number of posts about medical popular science that a doctor shares.	19.158	87.640
Paid feedback (*Paid*_*Feedback*_*it*_)	The cumulative number of virtual gifts that a doctor receives from patients.	122.477	279.942
Free feedback *(Free*_*Feedback*_*it*_)	The cumulative number of gratitude letters that a doctor receives from patients.	91.821	152.784
Online rating (*Rating*_*it*_)	The cumulative averaging rating that a doctor receives from patients.	4.737	0.226
Online tenure (*Tenure*_*it*_)	The number of days since a doctor opens the homepage.	2,018.483	976.137

### Empirical analysis

We employed a two-way panel fixed effects model (61) fully utilizing the panel data to reduce the effects of confounding factors while investigating the impacts of doctors' knowledge contributions and patient feedback on patients' effective use of OFUS. We used the doctor-fixed effects to control for time-invariant doctor-related factors (e.g., doctor's title, age, gender, other unobserved personality factors) and used the time-fixed effects to account for time-specific dynamics (e.g., seasonal factors, social policy changes). Based on the two-way panel fixed effects model, we estimated the following regression Equation (1):


(1) $$\begin{array}{l} {Effective\text{\_}Use}_{it}=\;\beta_{0}+{\beta_{1}Paid\text{\_}Contribution}_{it}+\\ {\beta_{2}Free\text{\_}Contribution}_{it}+{\beta_{3}Paid\text{\_}Feedback}_{it}+\beta_{4}Free\text{\_}\\ {Feedback}_{it}+\beta_{5}{Paid\text{\_}Contribution}_{it}\times {Free\text{\_}Contribution}_{it}+\\ \beta_{6}{Paid\text{\_}Feedback}_{it}\times {Free\text{\_}Feedback}_{it}+Controls+\\ \lambda_{i}+\mu_{t}+\varepsilon_{it} \end{array}$$

where *i* refers to a sampling doctor, and *t* refers to a month. *Effective*_*Use*_*it*_ is the dependent variable. Our independent variables include *Paid*_*Contribution*_*it*_, *Free*_*Contribution*_*it*_, *Paid*_*Feedback*_*it*_ and *Free*_*Feedback*_*it*_. The coefficients β_1_~β_4_ are the regression estimators of our main interest quantifying the effects of doctors' knowledge contributions and patient feedback, respectively, on patients' effective use of OFUS. λ_*i*_ refers to the doctor-fixed effects, and μ_*t*_ is the monthly fixed effects. *Controls* include the observed and time-variant control variables *Rating*_*it*_ and *Tenure*_*it*_. Finally, ε_*it*_ is the error term. We performed a log transformation for the dependent and independent variables due to their dispersed distributions. We report clustered-robust standard errors to reduce the potential issues of heteroskedasticity and serial correlation (62).

## Results

### Main results

We used ordinary least squares (OLS) to estimate the two-way fixed effects model. Table 2 presents the estimation results hierarchically. We first estimated model (1) with control variables and independent variables before adding the interaction terms, *Paid*_*Contribution*_*it*_ × *Free*_*Contribution*_*it*_ and *Paid*_*Feedback*_*it*_ × *Free*_*Feedback*_*it*_, to estimate model (2). To reduce multicollinearity concerns, we calculated the variance inflation factor (VIF) for our variables. We found that no VIF statistic is >5, which suggests the absence of multicollinearity concerns.

**Table 2:** Estimation results.

**Variables**	**(1)**	**(2)**
	**Model 1**	**Model 2**
*Paid*_*Contribution*_*it*_	0.245^***^	0.193^***^
	(0.018)	(0.019)
*Free*_*Contribution*_*it*_	0.187^***^	0.164^***^
	(0.025)	(0.058)
*Paid*_*Feedback*_*it*_	0.132^***^	0.343^***^
	(0.030)	(0.039)
*Free*_*Feedback*_*it*_	0.092^**^	0.306^***^
	(0.037)	(0.048)
*Paid_Contribution_it_* ^*^ *Free_Contribution_it_*		−0.004^*^
		(0.002)
*Paid_Feedback_it_* ^*^ *Free_Feedback_it_*		−0.067^***^
		(0.009)
*Rating*_*it*_	0.481^***^	0.483^***^
	(0.100)	(0.102)
*Tenure*_*it*_	−0.425^***^	−0.435^***^
	(0.046)	(0.046)
Constant	−0.393	−0.515
	(0.561)	(0.565)
Observations	91,165	91,165
*R*-squared	0.113	0.118
Number of doctors	3,672	3,672
Doctor FE	YES	YES
Time FE	YES	YES

Clustered-robust standard errors in parentheses. ^***^p < 0.01; ^**^p < 0.05; ^*^p < 0.1.

As shown in Column (1), the coefficients of both *Paid*_*Contribution*_*it*_ and *Free*_*Contribution*_*it*_ are significantly positive (β_1_ = 0.245, *p* < 0.01; β_2_ = 0.187, *p* < 0.01). This result implies that doctors' paid knowledge contributions and free knowledge contributions positively influence patients' effective use of OFUS, which supports hypothesis H1. Thus, when doctors have contributed more paid or free knowledge, their patients will be more likely to positively use their OFUS.

To test hypothesis H2, which predicts a difference in the influences of *Paid*_*Contribution*_*it*_ and *Free*_*Contribution*_*it*_, we first conducted a *T*-test for their coefficients. The testing results show that their betas are significantly different from each other (*p* < 0.01). Given that the coefficient magnitude of *Paid*_*Contribution*_*it*_ is higher than that of *Free*_*Contribution*_*it*_ (i.e., β_1_ > β_2_), doctors' paid knowledge contributions seem to have a stronger effect on patients' effective use of OFUS compared to free knowledge contributions. This finding supports hypothesis H2.

The results in Column (1) also show that both *Paid*_*Feedback*_*it*_ and *Free*_*Feedback*_*it*_ have positive and significant coefficients (β_3_ = 0.132, *p* < 0.01; β_4_ = 0.092, *p* < 0.05), which indicates that patients' paid and free feedback positively impact their effective use of OFUS. Specifically, when doctors receive more paid or free feedback, patients are more likely to positively use their OFUS. Meanwhile, the *T*-test results of their coefficients show that their betas are significantly different (*p* < 0.01). Given that the coefficient magnitude of *Paid*_*Feedback*_*it*_ is higher than that of *Free*_*Feedback*_*it*_ (i.e., β_3_ > β_4_), it appears that patients' paid feedback has a stronger effect on patients' effective use of OFUS compared to patients' free feedback. Hence, hypotheses H4 and H5 are supported.

Hypothesis H3 predicts a substitutional relationship between paid knowledge contributions and free knowledge contributions. As shown in Column (2), the coefficient of the interaction term *Paid*_*Contribution*_*it*_ × *Free*_*Contribution*_*it*_ is significant but negative (β_5_ = −0.004, *p* < 0.1), which suggests that doctors' paid knowledge contributions and free knowledge contributions have a substitutional relationship in influencing patients' effective use of OFUS. Furthermore, the strong central cue, paid knowledge contributions, negatively moderates the impact of the weak central cue, free knowledge contributions, on patients' effective use of OFUS. In other words, when doctors have provided a substantial amount of paid knowledge, the positive effect of free knowledge contributions on patients' effective use of OFUS will be weaker. Thus, hypothesis H3 is supported.

Similarly, the results in Column (2) show that the interaction term *Paid*_*Feedback*_*it*_ × *Free*_*Feedback*_*it*_ has a significant and negative coefficient (β_6_ = −0.067, *p* < 0.01), which indicates that patients' paid feedback and free feedback also have a substitutional relationship in influencing patients' effective use of OFUS. This result further suggests that paid feedback, a strong peripheral cue, has a negative moderating effect on the relationship between free feedback, a weak peripheral cue, and patients' effective use of OFUS. That is, the positive effect of free feedback on patients' effective use of OFUS will weaken as the amount of paid feedback from previous patients increases. Hypothesis H6 is therefore supported.

### Additional analyses

To test the robustness of our results, we conducted two additional robustness checks. First, since our dependent variable is a non-negative integer, we performed a count data model as a robustness check of our findings across model specifications. Given the excessive dispersion of the values of our dependent variable, we estimated a more suitable count model, namely a panel negative binomial regression model. Column (1) of Table 3 reports the estimated results, which are consistent with our main findings.

**Table 3:** The results of robustness checks.

**Variables**	**(1)**	**(2)**	**(3)**
	**Negative binomial regression**	**High-title doctors**	**Low-title doctors**
*Paid*_*Knowledge*_*it*_	0.310^***^	0.203^***^	0.255^***^
	(0.059)	(0.028)	(0.074)
*Free*_*Knowledge*_*it*_	0.158^***^	0.036^**^	0.190^***^
	(0.019)	(0.018)	(0.027)
*Paid*_*Feedback*_*it*_	0.377^***^	0.372^***^	0.359^***^
	(0.038)	(0.068)	(0.053)
*Free*_*Feedback*_*it*_	0.286^***^	0.307^***^	0.273^***^
	(0.042)	(0.059)	(0.071)
*Paid_Knowledge_it_* ^*^	−0.038^***^	−0.031^**^	−0.015^**^
*Free*_*Knowledge*_*it*_	(0.009)	(0.016)	(0.007)
*Paid_Feedback_it_* ^*^	−0.034^***^	−0.087^***^	−0.054^***^
*Free*_*Feedback*_*it*_	(0.008)	(0.012)	(0.012)
*Rating*_*it*_	0.843^***^	0.488^***^	0.444^***^
	(0.097)	(0.149)	(0.139)
*Tenure*_*it*_	−0.258^***^	−0.369^***^	−0.498^***^
	(0.039)	(0.068)	(0.061)
Constant	−5.283^***^	−0.925	0.059
	(0.545)	(0.855)	(0.760)
Observations	91,160	35,338	55,827
*R*-squared		0.135	0.106
Number of doctors	3,671	1,433	2,239
Doctor FE	YES	YES	YES
Time FE	YES	YES	YES

Clustered-robust standard errors in parentheses. ^***^p < 0.01; ^**^p < 0.05; ^*^p < 0.1.

Second, we performed a subsample analysis. In China, doctors' titles are usually divided into four ascending levels: resident doctors, attending doctors, deputy chief doctors, and chief doctors. A high-level title usually indicates that the doctor has extensive medical expertise and more service experience. According to the doctors' titles, we differentiated our overall sample into two groups: high-title doctors and low-title doctors. We regarded chief doctors as high-title doctors and the other doctors as low-title doctors (63). We then re-estimated our regression equations for both the high-title and low-title doctor samples. As shown in Columns (2) and (3) of Table 3, the results still support our main findings.

Third, in our main analysis, we mainly focused on the positive free feedback. However, in our research context, patients can provide negative feedback by posting negative online reviews. Therefore, we performed a supplement analysis to examine the impact of negative patient feedback (i.e., negative online reviews) on patients' effective use of OFUS. The estimated coefficient of negative patient feedback is significantly negative (β = −0.04, *p* < 0.05), which indicates that negative patient feedback discourages patients from effectively using OFUS.

## Discussion and implications

### Discussion

Based on the ELM, the current study has investigated the effects of doctors' knowledge contributions (paid and free) and patient feedback (paid and free) on patients' effective use of doctors' OFUS. We considered the doctors' knowledge contributions to be central cues processed through the central route and patient feedback to be peripheral cues processed through the peripheral route. The empirical findings of our analysis using a large set of panel data reveal important insights regarding patients' use behaviors of doctors' OFUS through online communication platforms.

First, our findings show that both doctors' knowledge contributions and patient feedback have a positive relationship with patients' effective use of doctors' OFUS, possibly because they are positive central and peripheral cues, respectively, of doctors' online service quality. Thus, doctors who have made extensive paid and free knowledge contributions or received more paid or free feedback from patients are more likely to persuade offline patients to positively use their OFUS. This finding is supported by the ELM literature stating that positive central cues and peripheral cues can positively influence individuals' behavior and decisions (11, 13). These findings are also similar to those of prior research on the effects of online information on patients' use decisions regarding doctors' online services. For example, Cao et al. (13) found that doctors' paid knowledge contributions for repeat patients had a positive impact on subsequent patients' choices of online consultation services, and Meng et al. (52) determined that doctors' free knowledge contributions encouraged potential patients to use their online consultation services. Similarly, in a study on patient feedback, Yang and Zhang (15) found that both paid and free feedback can positively influence subsequent patients' choices of online consultation services. Compared to these extant studies, which have mainly focused on one particular type of online information based on cross-sectional data, the current research has investigated a broader spectrum of online information within a single study, and our use of panel data provides more convincing empirical evidence that patients value different types of online information in their effective use decisions regarding OFUS.

Second, this study has found that doctors' paid knowledge contributions and patients' paid feedback have stronger effects on patients' effective use of OFUS compared to free knowledge contribution and free feedback, respectively. That is, paid doctors' knowledge contributions and paid patient feedback can better persuade patients to actively use OFUS compared to free knowledge contributions and free feedback. These differential effects demonstrate that paid knowledge contributions have more argument strength than free knowledge contributions, and the argument strength of paid feedback is stronger than that of free feedback. These findings can be explained by related ELM literature positing that a message with a strong argument often generates a greater persuasive effect than a message with a weak argument (11, 44). Furthermore, our findings can be supported by the signaling theory in management research, which dictates that signalers (e.g., doctors) can send signals that reflect their service quality to receivers (e.g., patients). After observing the signals from signalers, receivers can evaluate the service quality of the signalers and then take specific actions (e.g., purchasing or using a doctor's services). According to this theory, the strength and value of one signal are related to its cost (64). In the present study, both doctors' knowledge contributions and patient feedback can be considered important signals related to doctors' service quality. Therefore, paid knowledge contributions and paid feedback might have a higher signaling strength than free knowledge contribution and free feedback, respectively, because of the high-level financial cost. In addition, previous research has found that the effect of paid feedback on patients' choice of online consultation services is greater than the effect of free feedback (15), which supports our findings to some extent.

Third, in addition to the main effect, this study has investigated the relationship between different central cues (and different peripheral cues) in shaping patients' effective use of OFUS. The results show a substitutional relationship in influencing patients' effective use of OFUS between doctors' paid knowledge contributions and free knowledge contributions and between patients' paid feedback and free feedback. This finding might be related to the argument strength of the information cues and the information processing of individuals (11, 43). Since paid knowledge contributions have more argument strength than free knowledge contributions, we speculate that processing more paid knowledge contributions through the central route might lead to less processing of free knowledge contributions. Thus, the persuasive effect of paid knowledge contributions is likely to substitute that of free knowledge contributions in influencing patients' effective use of OFUS. Similarly, since paid feedback has more argument strength than free feedback, patients may rely primarily on paid feedback to make quick, lower-effort decisions through peripheral route processing, thus reducing their reliance on the information cues of free feedback. If so, to some extent, the persuasive effect of paid feedback is likely to substitute that of free feedback in influencing patients' effective use of OFUS. This conclusion is consistent with previous research suggesting that paid feedback and free feedback have a substitutional relationship in influencing patients' selection of online consultation services (15).

### Research implications

This study contributes to the literature on the ELM, medical follow-ups, and online healthcare services. While the ELM has been widely applied in health-related research (43, 46), the application of the ELM to online healthcare services and OFUS in particular is still in its infancy (13). Compared to previous research, the current study extends the persuasive outcomes in the ELM literature with an investigation of patients' effective use behaviors regarding OFUS. Furthermore, the results in our research context suggest that the argument strength of a message might be associated with its financial attribute, and a strong central (peripheral) cue might substitute the persuasive effects of a relatively weak central (peripheral) cue, which further enriches the findings in the extant literature on the ELM (11).

This study also adds to the literature on medical follow-ups and online healthcare services. Previous research on medical follow-ups has focused on offline and telephone follow-ups and mainly explored their effects on patients' health outcomes (16, 24). Although Li et al. (10) have investigated several online factors influencing patients' adoption of OFUS, research has rarely attempted to empirically examine patients' effective use of OFUS. Compared to a simple adoption behavior, effective use of OFUS is far more important for patients to effectively manage their health after offline diagnosis and treatment.

This study has key practical implications for OHC platforms and healthcare providers. First, the findings indicate that online information about both doctors' knowledge contributions and patient feedback positively influences patients' effective use of OFUS. Thus, OHC platforms should optimize their website design to display online information about doctors' knowledge contributions and patient feedback in a more user-friendly way that allows offline patients to gather more relevant information from doctors' homepages. This information can help patients learn more about doctors' OFUS, encourage them to better evaluate the target doctors and actively use their OFUS, and ultimately increase user traffic and active participation of patients on OHC platforms. Second, doctors who provide OFUS on OHC platforms should better manage their online information to highlight high medical service quality, attitude, and eWOM. For example, they can actively deliver paid health knowledge contributions through online consultation services, contribute more free health knowledge by posting health science education articles, and strive to elicit more paid and free feedback from patients. Through such efforts, doctors can promote active OFUS use among offline patients who have been treated and diagnosed by them in hospitals. This action will help realize long-term communication between doctors and patients and reduce strained relationships between them, especially in China. Moreover, offline patients who use doctors' OFUS are likely to be converted into consumers of the doctors' paid consultation services, which can further improve the economic return for doctors (52).

### Limitations and future directions

This study has several limitations that highlight directions for future research. First, we used empirical data deriving from only one OHC in China, which potentially limits the generalizability of our results. Future work should test our research model based on other OHCs in different cultural contexts. Second, we sampled doctors who specialize in chronic diseases, because of the high applicability of OFUS for patients with chronic diseases. However, it would be worthwhile for researchers to empirically evaluate our findings among doctors specialized in other diseases. Third, because of the unavailability of patient-level data, we constructed doctor-level panel data to examine patients' effective use of OFUS. Thus, the study does not include measures related to patient characteristics (e.g., health literacy, motivation, attitude). To further investigate the factors associated with patients and their impact, future studies can collect more relevant patient-level data via survey methods to better understand patients' effective use of OFUS. Finally, we measured patients' effective use of OFUS by observing whether a patient had at least one communication interaction with their doctor after adopting the doctor's OFUS. To comprehensively evaluate patients' effective use of OFUS, future research can perform content analyses on doctor-patient communication during the online follow-up process.

## Conclusion

The digitalization process is underway within many healthcare systems, including that of China. Online follow-up services in OHCs are a key component of this healthcare digitalization movement. However, since OFUS is a novel mode of medical follow-up communication, its effective use rate among patients is still relatively low. For OFUS to be effective, it is essential to explore online information factors that influence patients' effective use of OFUS. The present study has revealed that doctors' knowledge contributions (both paid and free) and patient feedback (both paid and free) can play important roles in persuading patients to effectively use OFUS. In sum, the findings of this study not only enrich the extant literature on medical follow-ups and online healthcare services but also provide valuable insights for OHC platforms and healthcare providers, who can better persuade patients to effectively use OFUS by optimizing communication strategies and can ultimately contribute to improving patients' health outcomes.

## Data availability statement

The raw data supporting the conclusions of this article will be made available by the authors, without undue reservation.

## Author contributions

SH: Conceptualization, Data curation, Formal analysis, Investigation, Methodology, Writing – original draft. LL: Conceptualization, Data curation, Formal analysis, Investigation, Methodology, Project administration, Software, Supervision, Writing – original draft, Writing – review & editing.

## Footnotes

^1^For details, please see https://www.haodf.com/info/aboutus.php.

## References

[1] Jackson C Shahsahebi M Wedlake T DuBard CA. Timeliness of outpatient follow-up: an evidence-based approach for planning after hospital discharge. Ann Fam Med. (2015) 13:115–22. 10.1370/afm.1753 25755032 PMC4369604

[2] Beaver K Tysver-Robinson D Campbell M Twomey M Williamson S Hindley A. Comparing hospital and telephone follow-up after treatment for breast cancer: randomised equivalence trial. BMJ. (2009) 338:a3147. 10.1136/bmj.a3147 19147478 PMC2628299

[3] Drewek R Mirea L Adelson PD. Lead time to appointment and no-show rates for new and follow-up patients in an ambulatory clinic. Health Care Manag. (2017) 36:4–9. 10.1097/HCM.0000000000000148 28067678

[4] Beaver K Williamson S Chalmers K. Telephone follow-up after treatment for breast cancer: views and experiences of patients and specialist breast care nurses. J Clin Nurs. (2010) 19:2916–24. 10.1111/j.1365-2702.2010.03197.x 20649914

[5] Zhao A Tang Q Gao Y. The effect of online follow-up services on offline and online physician demand: evidence from chronic disease physicians. In: PACIS 2023 Proceedings. (2023). p. 92. Available online at: https://aisel.aisnet.org/pacis2023/92 (accessed March 5, 2024).

[6] Liu PL Yeo TED. How online patient-provider communication impacts quality of life: examining the role of patient-centered care and health competence. Health Commun. (2023) 38:562–7. 10.1080/10410236.2021.1961971 34340609

[7] Jiang S. How does online patient–provider communication heal? Examining the role of patient satisfaction and communication experience in China. Health Commun. (2019) 34:1637–44. 10.1080/10410236.2018.1517634 30198772

[8] Gong Z Han Z Li X Yu C Reinhardt JD. Factors influencing the adoption of online health consultation services: the role of subjective norm, trust, perceived benefit, and offline habit. Front Public Health. (2019) 7:286. 10.3389/fpubh.2019.00286 31637229 PMC6787145

[9] Bao CZ Singh H Meyer B Kirksey K Bardhan I. Patient-provider engagement and its impact on health outcomes: A longitudinal study of patient portal use. Mis Quart. (2020) 44:699–723. 10.25300/MISQ/2020/14180

[10] Li C-R Zhang E Han J-T. Adoption of online follow-up service by patients: an empirical study based on the elaboration likelihood model. Comput Hum Behav. (2021) 114:106581. 10.1016/j.chb.2020.106581

[11] Petty RE Cacioppo JT. The Elaboration Likelihood Model of Persuasion. New York, NY: Springer (1986).

[12] Lord KR Lee M-S Sauer PL. The combined influence hypothesis: central and peripheral antecedents of attitude toward the ad. J Advertising. (1995) 24:73–85. 10.1080/00913367.1995.10673469

[13] Cao XY Liu Y Zhu Z Hu J Chen X. Online selection of a physician by patients: empirical study from elaboration likelihood perspective. Comput Hum Behav. (2017) 73:403–12. 10.1016/j.chb.2017.03.060

[14] Zhang X Guo F Xu T Li Y. What motivates physicians to share free health information on online health platforms? Inf Process Manag. (2020) 57:102166. 10.1016/j.ipm.2019.102166

[15] Yang HL Zhang XF. Investigating the effect of paid and free feedback about physicians' telemedicine services on patients' and physicians' behaviors: panel data analysis. J Med Int Res. (2019) 21:e12156. 10.2196/12156 30900997 PMC6450473

[16] Baker R Freeman GK Haggerty JL Bankart MJ Nockels KH. Primary medical care continuity and patient mortality: a systematic review. Brit J Gen Pract. (2020) 70:e600–11. 10.3399/bjgp20X712289 32784220 PMC7425204

[17] Kripalani S Theobald CN Anctil B Vasilevskis EE. Reducing hospital readmission rates: current strategies and future directions. Annu Rev Med. (2014) 65:471–85. 10.1146/annurev-med-022613-090415 24160939 PMC4104507

[18] Bible JE Shau DN Kay HF Cheng JS Aaronson OS Devin CJ. Are low patient satisfaction scores always due to the provider? Spine. (2018) 43:58–64. 10.1097/BRS.0000000000001453 26780613

[19] Sharp B Singal B Pulia M Fowler J Simmons S. You've got mail… and need follow-up: the effect and patient perception of E-mail follow-up reminders after emergency department discharge. Acad Emerg Med. (2015) 22:47–53. 10.1111/acem.12564 25546255

[20] Bigna JJR Noubiap JJN Kouanfack C Plottel CS Koulla-Shiro S. Effect of mobile phone reminders on follow-up medical care of children exposed to or infected with HIV in Cameroon (MORE CARE): a multicentre, single-blind, factorial, randomised controlled trial. Lancet Infect Dis. (2014) 14:600–8. 10.1016/S1473-3099(14)70741-8 24932893

[21] Liew S-M Tong SF Lee VKM Ng CJ Leong KC Teng CL. Text messaging reminders to reduce non-attendance in chronic disease follow-up: a clinical trial. Brit J Gen Pract. (2009) 59:916–20. 10.3399/bjgp09X472250 19712544 PMC2784529

[22] Lin H Wu X. Intervention strategies for improving patient adherence to follow-up in the era of mobile information technology: a systematic review and meta-analysis. PLoS ONE. (2014) 9:e104266. 10.1371/journal.pone.0104266 25100267 PMC4123963

[23] Garnett WR Davis LJ McKenney JM Steiner KC. Effect of telephone follow-up on medication compliance. Am J Hosp Pharm. (1981) 38:676–9. 10.1093/ajhp/38.5.676 7282695

[24] Arad M Goli R Parizad N Vahabzadeh D Baghaei R. Do the patient education program and nurse-led telephone follow-up improve treatment adherence in hemodialysis patients? A randomized controlled trial. BMC Nephrol. (2021) 22:1–13. 10.1186/s12882-021-02319-9 33827478 PMC8028152

[25] Turner D. Can telephone follow-up improve post-discharge outcomes? Br J Nurs. (1996) 5:1361–5. 10.12968/bjon.1996.5.22.1361 9025364

[26] Braun E Baidusi A Alroy G Azzam ZS. Telephone follow-up improves patients satisfaction following hospital discharge. Eur J Intern Med. (2009) 20:221–5. 10.1016/j.ejim.2008.07.021 19327616

[27] Booker J Eardley A Cowan R Logue J Wylie J Caress A-L. Telephone first post-intervention follow-up for men who have had radical radiotherapy to the prostate: evaluation of a novel service delivery approach. Eur J Oncol Nurs. (2004) 8:325–33. 10.1016/j.ejon.2004.01.003 15550362

[28] Lopez-Villegas A Catalan-Matamoros D Peiro S Lappegard KT Lopez-Liria R. Cost–utility analysis of telemonitoring versus conventional hospital-based follow-up of patients with pacemakers. The NORDLAND randomized clinical trial. PLoS ONE. (2020) 15:e0226188. 10.1371/journal.pone.0226188 31995558 PMC6988929

[29] Naik N Hameed B Sooriyaperakasam N Vinayahalingam S Patil V Smriti K. Transforming healthcare through a digital revolution: a review of digital healthcare technologies and solutions. Front Digit Health. (2022) 4:919985. 10.3389/fdgth.2022.919985 35990014 PMC9385947

[30] Huang N Yan Z Yin H. Effects of online–offline service integration on e-healthcare providers: a quasi-natural experiment. Prod Oper Manag. (2021) 30:2359–78. 10.1111/poms.13381

[31] Yang HL Guo XT Wu TS Ju XF. Exploring the effects of patient-generated and system-generated information on patients' online search, evaluation and decision. Electron Commer Res Appl. (2015) 14:192–203. 10.1016/j.elerap.2015.04.001

[32] Chen J Hsu P-Y Chang Y-W Shiau W-L Lan Y-C. For free or paid? A comparison of doctors' intention to offer consulting services in eHealth. Ind Manage Data Syst. (2022) 122:1816–52. 10.1108/IMDS-05-2021-0336

[33] Yang M Jiang J Cameron A-F Liu X. How do you cope? Online medical consultation service uncertainty, coping strategies, and subsequent payment. Electron Commer Res Appl. (2023) 61:101294. 10.1016/j.elerap.2023.101294

[34] Meng F Liu Y Zhang X Liu L. General knowledge-sharing and patient engagement in online health communities: an inverted U-shaped relationship. J Knowl Manag. (2023) 28:763–88. 10.1108/JKM-12-2022-0986

[35] Wu QL Tang L. What satisfies parents of pediatric patients in China: a grounded theory building analysis of online physician reviews. Health Commun. (2022) 37:1329–36. 10.1080/10410236.2021.1888437 33601987

[36] Xu YQ Armony M Ghose A. The interplay between online reviews and physician demand: an empirical investigation. Manage Sci. (2021) 67:7344–61. 10.1287/mnsc.2020.3879

[37] Wang J-J Liu H Cui X Ye J Chen H. Impact of a physician's prosocial behavior on the patient's choice: an empirical investigation in online health community. Inform Technol Peopl. (2023) 36:1703–25. 10.1108/ITP-12-2020-0878

[38] Chen Q Jin J Zhang T Yan X. The effects of log-in behaviors and web reviews on patient consultation in online health communities: longitudinal study. J Med Internet Res. (2021) 23:e25367. 10.2196/25367 34081008 PMC8212624

[39] Liu X Guo X Wu H Wu T. The impact of individual and organizational reputation on physicians' appointments online. Int J Electron Comm. (2016) 20:551–77. 10.1080/10864415.2016.1171977

[40] Liu F Li Y Ju X. Exploring patients' consultation behaviors in the online health community: the role of disease risk. Telemed E-Health. (2019) 25:213–20. 10.1089/tmj.2018.0033 29927721

[41] Lu W Wu H. How online reviews and services affect physician outpatient visits: content analysis of evidence from two online health care communities. JMIR Med Inform. (2019) 7:373–94. 10.2196/preprints.16185 31789597 PMC6915441

[42] Angst CM Agarwal R. Adoption of electronic health records in the presence of privacy concerns: the elaboration likelihood model and individual persuasion. Mis Quart. (2009) 33:339–70. 10.2307/20650295

[43] Petty RE Barden J Wheeler SC. The elaboration likelihood model of persuasion: developing health promotions for sustained behavioral change. Emerg Theories Health Promot Pract Res. (2009) 2:185–214.

[44] Kononova A Yuan S Joo E. Reading about the flu online: How health-protective behavioral intentions are influenced by media multitasking, polychronicity, and strength of health-related arguments. Health Commun. (2017) 32:759–67. 10.1080/10410236.2016.1172289 27419820

[45] Chen Y Yang L Zhang M Yang J. Central or peripheral? Cognition elaboration cues' effect on users' continuance intention of mobile health applications in the developing markets. Int J Med Inform. (2018) 116:33–45. 10.1016/j.ijmedinf.2018.04.008 29887233

[46] Jin J Yan X Li Y Li Y. How users adopt healthcare information: an empirical study of an online Q&A community. Int J Med Inform. (2016) 86:91–103. 10.1016/j.ijmedinf.2015.11.002 26616406

[47] Chaiken S. Heuristic versus systematic information processing and the use of source versus message cues in persuasion. J Pers Soc Psychol. (1980) 39:752–66. 10.1037/0022-3514.39.5.752

[48] Petty RE Cacioppo JT. The effects of involvement on responses to argument quantity and quality: Central and peripheral routes to persuasion. J Pers Soc Psychol. (1984) 46:69. 10.1037//0022-3514.46.1.69

[49] Slater MD Rouner D. How message evaluation and source attributes may influence credibility assessment and belief change. J Mass Commun Q. (1996) 73:974–91. 10.1177/107769909607300415

[50] Youk S Malik M Chen Y Hopp FR Weber R. Measures of argument strength: a computational, large-scale analysis of effective persuasion in real-world debates. Commun Methods Meas. (2024) 18:7–29. 10.1080/19312458.2023.2230866

[51] Johnson BT Eagly AH. Effects of involvement on persuasion: a meta-analysis. Psychol Bull. (1989) 106:290–314. 10.1037//0033-2909.106.2.290

[52] Meng F Zhang X Liu L Ren C. Converting readers to patients? From free to paid knowledge-sharing in online health communities. Inf Process Manag. (2021) 58:102490. 10.1016/j.ipm.2021.102490

[53] Chowdhury RM Finn A Olsen GD. Investigating the simultaneous presentation of advertising and television programming. J Advert. (2007) 36:85–96. 10.2753/JOA0091-3367360306

[54] Wang S Cunningham NR Eastin MS. The impact of eWOM message characteristics on the perceived effectiveness of online consumer reviews. J Inter Advert. (2015) 15:151–9. 10.1080/15252019.2015.1091755

[55] Park D-H Kim S. The effects of consumer knowledge on message processing of electronic word-of-mouth via online consumer reviews. Electron Commer Res Appl. (2008) 7:399–410. 10.1016/j.elerap.2007.12.001

[56] Teng S Wei Khong K Wei Goh W Yee Loong Chong A. Examining the antecedents of persuasive eWOM messages in social media. Online Inform Rev. (2014) 38:746–68. 10.1108/OIR-04-2014-0089

[57] Cheung CM Lee MK Rabjohn N. The impact of electronic word-of-mouth: the adoption of online opinions in online customer communities. Internet Res. (2008) 18:229–47. 10.1108/10662240810883290

[58] Peng L Wang Y Chen J. Consequences of gift giving in online health communities on physician service quality: empirical text mining study. J Med Int Res. (2020) 22:e18569. 10.2196/18569 32729834 PMC7426794

[59] Wang Y Wu H Xia C Lu N. Impact of the price of gifts from patients on physicians' service quality in online consultations: empirical study based on social exchange theory. J Med Internet Res. (2020) 22:e15685. 10.2196/15685 32369028 PMC7238091

[60] Li Y Ma X Song J Yang Y Ju X. Exploring the effects of online rating and the activeness of physicians on the number of patients in an online health community. Telemed EHealth. (2019) 25:1090–8. 10.1089/tmj.2018.0192 30676279

[61] Gardiner JC Luo Z Roman LA. Fixed effects, random effects and GEE: what are the differences? Stat Med. (2009) 28:221–39. 10.1002/sim.3478 19012297

[62] Barasa L Knoben J Vermeulen P Kimuyu P Kinyanjui B. Institutions, resources and innovation in East Africa: a firm level approach. Res Policy. (2017) 46:280–91. 10.1016/j.respol.2016.11.008

[63] Fan WJ Zhou QQ Qiu LF Kumar S. Should doctors open online consultation services? An empirical investigation of their impact on offline appointments. Inf Syst Res. (2023) 34:629–51. 10.1287/isre.2022.1145

[64] Connelly BL Certo ST Ireland RD Reutzel CR. Signaling theory: a review and assessment. J Manage. (2011) 37:39–67. 10.1177/0149206310388419

